# Molecular evolution of *rbcL *in three gymnosperm families: identifying adaptive and coevolutionary patterns

**DOI:** 10.1186/1745-6150-6-29

**Published:** 2011-06-03

**Authors:** Lin Sen, Mario A Fares, Bo Liang, Lei Gao, Bo Wang, Ting Wang, Ying-Juan Su

**Affiliations:** 1 CAS Key Laboratory of Plant Germplasm Enhancement and Specialty Agriculture, Wuhan Botanical Garden, Chinese Academy of Sciences, Wuhan, China; 2 Graduate University of Chinese Academy of Sciences, Beijing, China; 3 Evolutionary Genetics and Bioinformatics Laboratory, Department of Genetics, Smurfit Institute of Genetics, University of Dublin, Trinity College Dublin, Dublin 2, Ireland; 4 Integrative and Systems Biology Group, Department of Abiotic Stress, Instituto de Biologia Molecular y Celular de Plantas (CSIC-Universidad Politecnica de Valencia), Valencia, Spain; 5 Department of Biochemistry, University of Missouri, Columbia, USA; 6 Genetics Area Program, University of Missouri, Columbia, USA; 7 State Key Laboratory of Biocontrol, School of Life Sciences, Sun Yat-Sen University, Guangzhou, China

## Abstract

**Background:**

The chloroplast-localized ribulose-1, 5-biphosphate carboxylase/oxygenase (Rubisco), the primary enzyme responsible for autotrophy, is instrumental in the continual adaptation of plants to variations in the concentrations of CO_2_. The large subunit (LSU) of Rubisco is encoded by the chloroplast *rbcL *gene. Although adaptive processes have been previously identified at this gene, characterizing the relationships between the mutational dynamics at the protein level may yield clues on the biological meaning of such adaptive processes. The role of such coevolutionary dynamics in the continual fine-tuning of RbcL remains obscure.

**Results:**

We used the timescale and phylogenetic analyses to investigate and search for processes of adaptive evolution in *rbcL *gene in three gymnosperm families, namely Podocarpaceae, Taxaceae and Cephalotaxaceae. To understand the relationships between regions identified as having evolved under adaptive evolution, we performed coevolutionary analyses using the software CAPS. Importantly, adaptive processes were identified at amino acid sites located on the contact regions among the Rubisco subunits and on the interface between Rubisco and its activase. Adaptive amino acid replacements at these regions may have optimized the holoenzyme activity. This hypothesis was pinpointed by evidence originated from our analysis of coevolution that supported the correlated evolution between Rubisco and its activase. Interestingly, the correlated adaptive processes between both these proteins have paralleled the geological variation history of the concentration of atmospheric CO_2_.

**Conclusions:**

The gene *rbcL *has experienced bursts of adaptations in response to the changing concentration of CO_2 _in the atmosphere. These adaptations have emerged as a result of a continuous dynamic of mutations, many of which may have involved innovation of functional Rubisco features. Analysis of the protein structure and the functional implications of such mutations put forward the conclusion that this evolutionary scenario has been possible through a complex interplay between adaptive mutations, often structurally destabilizing, and compensatory mutations. Our results unearth patterns of evolution that have likely optimized the Rubisco activity and uncover mutational dynamics useful in the molecular engineering of enzymatic activities.

**Reviewers:**

This article was reviewed by Prof. Christian Blouin (nominated by Dr W Ford Doolittle), Dr Endre Barta (nominated by Dr Sandor Pongor), and Dr Nicolas Galtier.

## Background

In spite of its slow non-specific catalysis the chloroplast-localized ribulose-1,5-biphosphate carboxylase/oxygenase (Rubisco, EC 4.1.1.39), the most abundant protein in nature, is the primary enzyme responsible for autotrophy [1]. Rubisco is a bi-functional enzyme catalyzing both the carboxylation of D-ribulose-1,5-bisphosphate (RuBP) that initiates photosynthetic CO_2 _fixation and the oxygenation of RuBP that starts the nonessential photo-respiratory pathway [2]. Its holoenzyme in green algae and higher plants consists of eight large subunits (LSUs) encoded by the chloroplast (cp) gene *rbcL *and eight small subunits (SSUs) encoded by the nuclear gene *rbcS*. Active sites are formed at the intra-dimer interfaces among the C-terminal, α/β barrel domain of one large subunit and the N-terminal domain of another [3]. CO_2 _and O_2 _mutually compete as substrates for the active sites, and the ratio of carboxylation to oxygenation ultimately affects the efficiency of net carbon assimilation [4]. Understanding of the molecular evolution of *rbcL *genes may shed light on the functional/structural features governing Rubisco activity. The knowledge is also paramount from the biotechnological point of view since photosynthesis is tightly linked to the delicate balance between carboxylation and oxygenation. Subtle alteration of this balance can have a significant impact upon photosynthetic productivity [5].

Most of the evolutionary changes optimizing Rubisco's function have been likely subjected to selection forces owing to the direct relationship between this function and the biological fitness of the plant. Most molecular changes favouring the enzyme function would be fixed by adaptive evolution, while the changes compromising its activity would be removed by purifying selection. Following the inverse rational, investigation of adaptive evolution processes in Rubisco can aid in identifying key changes in its function. Adaptive evolution has seldom been observed in cp genes [6], mainly due to: i) Low rates of evolution of protein-coding cp genes [7], which challenges the sensitivity of the methods to identify selection due to the lack of statistical power [8]; ii) With the exception of few cases [6], cp genes present a low propensity to undergo duplication [9], a fundamental mechanism in generating the source of novel functions [10]; and iii) The lack of models that realistically parameterize the evolution of cp genes, known to present codon usage bias and hence to yield misleading selection results when inappropriate models are applied [11,12]. Nonetheless, the use of more realistic models may prove successful in identifying true adaptive molecular changes in cp genes, many of which would be linked to plant adaptive radiations [13].

Rubisco enzymatic efficiency has been shown to be fine-tuned in diverse autotrophy species [14]. Significant positive selection events have been also identified in the RbcL subunit of most land plant lineages by using simulation approaches [15]. In particular, the adaptive evolution of *rbcL *genes has been found in the aquatic plant *Potamogeton *[16] and the F-type lineage of *Conocephalum *[17]. These findings make it plausible to hypothesize that RbcL subunit may have undergone "continual fine-tuning" in green plants to adapt to CO_2 _concentration changes across geological epochs [18]. How are these adaptive process reflected at the molecular level? To understand and answer these questions, adaptive evolution analysis must be complemented with the identification of coevolutionary dynamics that can highlight the intricate co-adaptive relationships between residues in the protein under an estimated timescale [14,19]. In this respect, current molecular evolutionary methods such as the identification of coevolutionary sites [20] and relaxed molecular clock inference models [21,22] offer a unique opportunity to dissect the *rbcL *gene fine-tuning.

Podocarpaceae comprises members that extend both southern and northern hemispheres, accounting for nearly 14% of the gymnosperm diversity. The family is predominantly occupying mesic temperate and sunny tropical mountain habitats [23]. Taxaceae (also known as taxads or the yew family), including 25 species, is a widespread albeit locally endangered gymnosperm family [24]. The genus *Cephalotaxus *was previously listed in the Taxaceae [25]. However, recent molecular evidences confirmed that it should be treated as a family (Cephalotaxaceae) [26]. Since these three families possess different contemporary net diversification, characterizations of the evolutionary processes in key genes may help to unearth the dynamics of their species diversification. In the present study we conduct an exhaustive analysis of such evolutionary dynamics and address the following questions: i) Whether and to what extent have adaptive evolution played a role in the evolutionary innovation of the *rbcL *gene; ii) How important have this evolutionary force been in the ecological diversification of the three gymnosperm families (Taxaceae, Cephalotaxaceae and Podocarpaceae); and iii) What is the coevolutionary pattern within the RbcL subunit of these families?

## Results

### Alignments and sequence characteristics

The alignment comprised 1269 positions (423 codons) of the *rbcL *gene. Gaps were excluded from the sequences. The model most suited to explain the substitution dynamics and the phylogenetic tree relating all the sequences of this multiple sequence alignment (MSA) was determined by Modeltest 3.7 [27] and Datamonkey 2010 website (http://www.datamonkey.org) [28].

### Dating the phylogenetic divergence events

**Figure 1:**
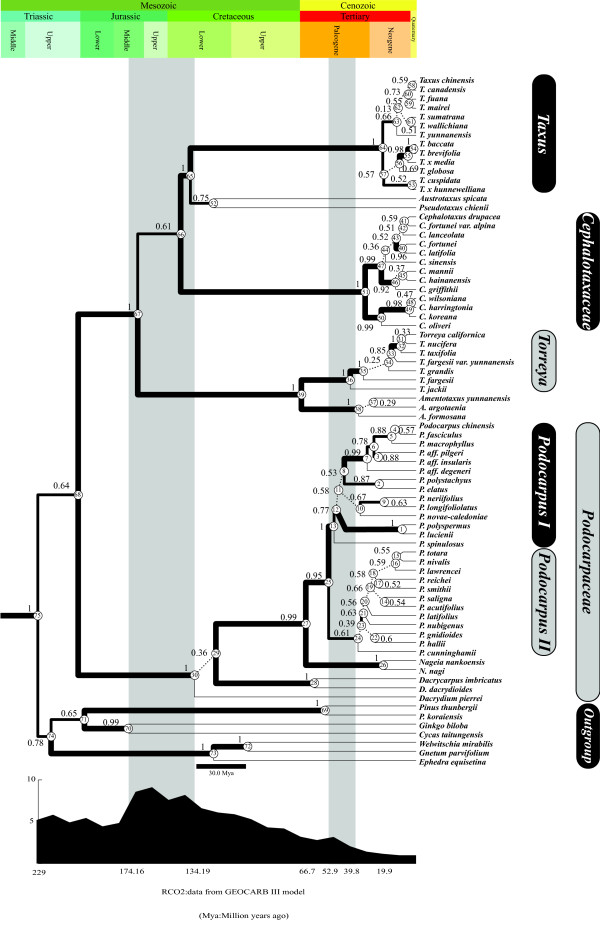
**The phylogenetic tree inferred from *rbcL *sequences under the UCLD model**. Geologic timescale is tagged above the phylogenetic tree (Unit: Million years). The estimated global atmospheric CO_2 _concentrations from the GEOCARB III model are mapped under the phylogenetic tree according to the geologic timeline. Each node in the tree is numbered. Posterior probability values are shown along the branches and those with posterior probability ≥ 0.9 are heavily thickened. Two time intervals are demonstrated in grey. The six major clades concerned in this research are indicated: Podocarpaceae, *Podocarpus *I and II, Cephalotaxaceae, *Taxus *and *Torreya*. The length of each branch is in proportion to the divergence time estimated by using the UCLD model.

Two monophyletic clades of Taxaceae-Cephalotaxaceae and Podocarpaceae were identified by the Bayesian analysis, both supported with high posterior probabilities (Figure 1). Within Podocarpaceae, besides *Podocarpus *three well-supported genera were obtained: *Nageia, Dacrycarpus *and *Dacrydium*. *Dacrydium *was basal to the remain Podocarpaceae species. The genus *Podocarpus *diverged into two groups, *Podocarpus *I and *Podocarpus *II. In the Taxaceae-Cephalotaxaceae clade, four major lineages were resolved. Among them the *Torreya-Amentotaxus *lineage was sister to the remainder; the family Cephalotaxaceae was sister to the three remaining genera of the family Taxaceae; and the genus *Taxus *was the sister to the *Pseudotaxus-Austrotaxus *lineage. These results indicated that the divergence between Taxaceae-Cephalotaxaceae and Podocarpaceae represented the first major split during the evolution of the three families and that Cephalotaxaceae is nested in Taxaceae.

The absolute divergence time and evolutionary rates of all nodes in the tree were estimated under the uncorrelated lognormal model (UCLD), and the global atmospheric CO_2 _concentrations were also mapped according to the estimated timescale (Figure 1, Table 1). This analysis provided a time estimate of 204 Mya to the most recent common ancestor (MRCA) for the extant Podocarpaceae (Figure 1). The results demonstrated that the splitting between the genera *Nageia *and *Podocarpus *occurred during early Paleocene following the Cretaceous-Tertiary (K/T) extinction event, after which the genus *Podocarpus *segregated into two groups (*Podocarpus *I and II). The lineage *Torreya*-*Amentotaxus *diverged from the remainder of the other members in Taxaceae-Cephalotaxaceae during the Jurassic; Cephalotaxaceae departed with the rest three genera in Taxaceae during the Cretaceous; and *Taxus *split with *Pseudotaxus-Austrotaxus *during the Tertiary.

**Table 1:** Parameters estimated under the UCLD model

**Node No**.	Posterior probability	Estimated divergence time (Mya)	Estimated rates **(10**^**-10 **^**nt/year)**	Geologic period
1	100	8.62	1.52	Neogene
7	99.97	29.61	2.85	Paleogene/Neogene
13	100	49.65	2.69	Paleogene
25	95.51	52.95	1.69	Paleogene
26	100	19.98	2.06	Neogene
27	99.99	66.75	2.46	Paleogene
28	99.99	61.78	1.99	Paleogene
30	100	134.19	4.66	Lower Cretaceous
32	100	9.13	2.41	Paleogene
35	99.67	31.9	1.96	Paleogene
36	100	39.88	2.94	Paleogene
38	100	34.74	1.28	Paleogene
39	99.99	69.45	1.65	Cretaceous/Paleogene
40	96.18	8.18	1.59	Neogene
47	99.29	21.02	1.56	Neogene
49	98.09	4.12	1.48	Neogene/Quaternary
50	98.77	21.05	1.52	Neogene
51	100	30.85	2.53	Paleogene/Neogene
54	98.18	1.47	2.01	Neogene/Quaternary
55	100	9.98	2.47	Neogene/Quaternary
64	100	20.17	2.57	Neogene
65	99.76	136.64	1.58	Lower Cretaceous
67	100	168.52	2.89	Middle Jurassic
70	99.97	174.16	1.97	Middle Jurassic

Results for the well supported nodes in Figure 1 are shown.

### Consistent adaptive evolution in the phylogeny of *rbcL*

**Figure 2:**
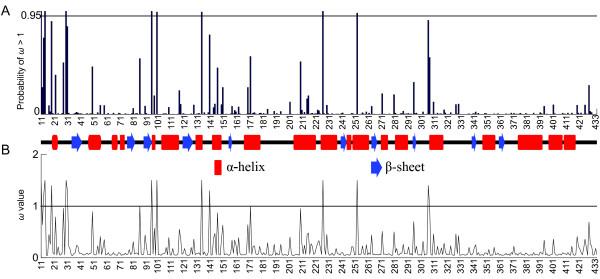
**Estimated parameters under the M8 model using Selecton web-server**. Approximate posterior means of *ω *are weighted by the posterior probabilities. Sites are numbered according to the reference sequence from *Taxus mairei *(GenBank accession number: AY450856).

The primary proportion of the sites (*p*_*0 *_> 89%, *ω*_0 _< 0.1) in the three families were under purifying selection (Figure 2, Table 2). A small proportion of sites (*p*_2 _= 2%, *ω*_2 _= 2.92) were under positive selection (Table 2). Seven sites (A11V, Q14K, K30Q, S95N, V99A, I133L, and L225I) were significantly identified as positively selected sites under both PAML and Selecton programs via random-site models, and three more (G86D, S143A, and T262V) were found to have evolved under adaptive evolution using the branch-site models. One site (K30Q) was also recognized as positively selected via conservative nested models (M1a/M2a). The significant level of nested models was assessed by the likelihood ratio test (Table 2). The χ^2 ^test indicated that all the alternative hypotheses (M2a, M3, and M8) in the random-site models significantly outperformed their comparable null test (M1a, M0, and M7/M8a). Moreover, the branch-site model A (*ω*_2 _estimated) fit five branches (Podocarpaceae, *Podocarpus *I and II, Taxaceae, and *Taxus*) significantly better than its null model (*ω*_2 _= 1 fixed) (Table 2 and 3). Podocarpaceae and *Podocarpus *I and II were also identified to be under positive selection, even after correcting for multiple tests by the Bonferroni correction [29] (Table 3). By contrast, when *Torreya *and Cephalotaxaceae were specified as the foreground branches, both the test and null models had similar log-likelihood values, indicating that there was not enough evidence to support adaptive diversifying scenarios.

**Table 2:** Parameter estimates from tests for selection

Model	*np*	ℓ	Parameters	Positively selected sites
M0: One ratio	137	-5654.77	***ω ***= 0.173	None
M1a: Nearly neutral	138	-5539.34	***p***_**0 **_= 0.885, ***ω***_**0 **_= 0.062; ***p***_**1 **_= 0.114, ***ω***_**1 **_= 1	Not allowed
M2a: Positive selection	140	-5529.73	***p***_**0 **_= 0.892, ***ω***_**0 **_= 0.07; ***p***_**0 **_= 0.085, ***ω***_**1 **_= 1; ***p***_**2 **_= 0.023, ***ω***_**2 **_= 2.92	K30Q
M3: Discrete	139	-5536.84	***p***_**0 **_= 0.92, ***ω***_**0 **_= 0.08; ***p***_**1 **_= 0.079, ***ω***_**1 **_= 1.46	A11V, Q14K, E28Q, K30Q, G86D, S95N, V99A, I133L, L225I, I251M, K305R
M7: β	138	-5547.37	***p ***= 0.015, ***q ***= 0.074	Not allowed
M8: β &*ω*	140	-5527.82	***p***_**0 **_= 0.96; ***p ***= 0.09, ***q ***= 0.38; ***p***_**1 **_= 0.039, ***ω ***= 2.20	A11V, Q14K, K30Q, S95N, V99A, I133L, L225I

**Branch-site models**				

**Taxaceae-Cephalotaxaceae**				

Model A: ***ω***_**2 **_= 1, fixed	139	-5533.19	***p***_**0**_***=***0.857***, ω***_**0**_***=***0.053;***p***_**1**_***=***0.061,***ω***_**1**_***=***1;***p***_**2a**_+***p***_**2b **_= 0.082, ***ω***_**2 **_= 1	Not allowed
Model A: ***ω***_**2 **_estimated	140	-5530.95	***p***_**0**_***=***0.891***, ω***_**0**_***=***0.06;***p***_**1**_***=***0.078,***ω***_**1**_***=***1;***p***_**2a**_+***p***_**2b **_= 0.03, ***ω***_**2 **_= 2.55	A11V, V99A, I133L

***Taxus***				

Model A: ***ω***_**2 **_= 1, fixed	139	-5535.98	***p***_**0**_***=***0.852***, ω***_**0**_***=***0.057;***p***_**1**_***=***0.106,***ω***_**1**_***=***1;***p***_**2a**_+***p***_**2b **_= 0.042, ***ω***_**2 **_= 1	Not allowed
Model A: ***ω***_**2 **_estimated	140	-5533.12	***p***_**0**_***=***0.86***, ω***_**0**_***=***0.06;***p***_**1**_***=***0.103,***ω***_**1**_***=***1;***p***_**2a**_+***p***_**2b **_= 0.027, ***ω***_**2 **_= 5.9	K30Q

***Torreya***				

Model A: ***ω***_**2 **_= 1, fixed	139	-5538.23	***p***_**0**_***=***0.82***, ω***_**0**_***=***0.059;***p***_**1**_***=***0.106,***ω***_**1**_***=***1;***p***_**2a**_+***p***_**2b **_= 0.073, ***ω***_**2 **_= 1	Not allowed
Model A: ***ω***_**2 **_estimated	140	-5538.33	***p***_**0**_***=***0.82***, ω***_**0**_***= ***0.059;***p***_**1**_***=***0.106,***ω***_**1**_***=***1;***p***_**2a**_+***p***_**2b **_= 0.071, ***ω***_**2 **_= 1.02	None

**Podocarpaceae**				

Model A: ***ω***_**2 **_= 1, fixed	139	-5527.9	***p***_**0**_***=***0.84***, ω***_**0**_***=***0.049;***p***_**1**_***=***0.057,***ω***_**1**_***=***1;***p***_**2a**_+***p***_**2b **_= 0.099, ***ω***_**2 **_= 1	Not allowed
Model A: ***ω***_**2 **_estimated	140	-5523.16	***p***_**0**_***=***0.89***, ω***_**0**_***=***0.065;***p***_**1**_***=***0.063,***ω***_**1**_***=***1;***p***_**2a**_+***p***_**2b **_= 0.047, ***ω***_**2 **_= 2.41	A11V, G86D, V99A, I133L

***Podocarpus *I**				

Model A: ***ω***_**2 **_= 1, fixed	139	-5537.56	***p***_**0**_***=***0.786***, ω***_**0**_***=***0.05;***p***_**1**_***=***0.084,***ω***_**1**_***=***1;***p***_**2a**_+***p***_**2b **_= 0.129, ***ω***_**2 **_= 1	Not allowed
Model A: ***ω***_**2 **_estimated	140	-5533.91	***p***_**0**_***=***0.727***, ω***_**0**_***=***0.058;***p***_**1**_***=***0.091,***ω***_**1**_***=***1;***p***_**2a**_+***p***_**2b **_= 0.181, ***ω***_**2 **_= 1.12	None

***Podocarpus *II**				

Model A: ***ω***_**2 **_= 1, fixed	139	-5530.31	***p***_**0**_***=***0.81***, ω***_**0**_***=***0.054;***p***_**1**_***=***0.093,***ω***_**1**_***=***1;***p***_**2a**_+***p***_**2b **_= 0.097, ***ω***_**2 **_= 1	Not allowed
Model A: ***ω***_**2 **_estimated	140	-5526.95	***p***_**0**_***=***0.85***, ω***_**0**_***=***0.056;***p***_**1**_***=***0.096,***ω***_**1**_***=***1;***p***_**2a**_+***p***_**2b **_= 0.056, ***ω***_**2 **_= 3.31	S143A, T262V

**Model**	***np***	ℓ	**Parameters**	**Positively selected sites**

**Cephalotaxaceae**				

Model A: ***ω***_**2 **_= 1, fixed	139	-5537.3	***p***_**0**_***=***0.82***, ω***_**0**_***=***0.058;***p***_**1**_***=***0.102,***ω***_**1**_***=***1;***p***_**2a**_+***p***_**2b **_= 0.077, ***ω***_**2 **_= 1	Not allowed
Model A: ***ω***_**2 **_estimated	140	-5536.84	***p***_**0**_***=***0.862***, ω***_**0**_***=***0.059;***p***_**1**_***=***0.105,***ω***_**1**_***=***1;***p***_**2a**_+***p***_**2b **_= 0.032, ***ω***_**2 **_= 2.68	None

Positive selection sites are identified with posterior probability higher than 95%.

**Table 3:** Tests for selection with Bonferroni correction

Model	2Δℓ	*d.f.*	*p*	Sig. α = 0.05	Bonferroni correction	Sig. α = 0.05
M0-M3	235.86	2	0	●	No need	
M1a-M2a	19.22	2	0.0001	●	No need	
M7-M8	39.1	2	0	●	No need	
M8a-M8	4.86	1	0.0275	●	No need	

A-A1 test						
Taxaceae-Cephalotaxaceae	4.48	1	0.0343	●	0.0686	○
*Taxus*	5.72	1	0.0168	●	0.084	○
*Torreya*	0.2	1	0.6547	○	3.2735	○
Cephalotaxaceae	0.92	1	0.3375	○	1.6875	○
Podocarpaceae	9.48	1	0.0021	●	0.0105	●
*Podocarpus *I	7.3	1	0.0069	●	0.0345	●
*Podocarpus *II	6.72	1	0.0095	●	0.0475	●

In the branch-site model, the relevant hypotheses are verified under the Bonferroni correction to avoid false positive conclusion. The sign ● denotes the results that pass the statistical significance tests, while ○ stands for the opposite.

**Figure 3:**
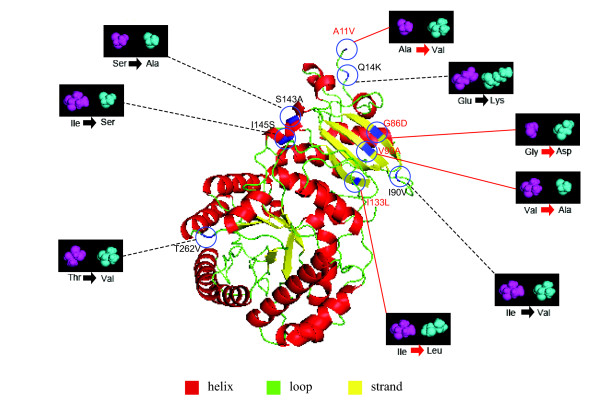
**Positively selected sites in the RbcL subunit of the Podocarpaceae ancestor**. Four positive selected sites of the Podocarpaceae ancestor are highlighted in red arrows. The 3D imagines of the ancestral and current amino acid residues are represented in purple and cyanine, respectively. The ancestral amino acid residues are inferred by the DAMBE package and the ancestral state reconstruction (ASR) molecule on Datamonkey 2010 website for the Podocarpaceae ancestral node [28,82]. The three domains are colour coded differently. Positively selected sites are indicated with lines, whereas the potential ones are with black dotted lines.

Four sites (A11V, G86D, V99A, and I133L) were detected under adaptive evolution along the ancestral branch of Podocarpaceae (Figure 3). The ancestor of *Podocarpus *I presented two positively selected candidate sites (A11V and Q14K); however, neither of them was significantly supported by the posterior probabilities. As for *Podocarpus *II, two sites (S143A and T262V) were identified under selection with strong statistical supports. In addition, three sites (A11V, V99A, and I133L) were found to be positively selected on the branch leading to the ancestor of Taxaceae, whereas only one (K30Q) was identified in the ancestor of *Taxus*.

### Inter-dependent evolution of amino acid sites in the RbcL subunit

**Figure 4:**
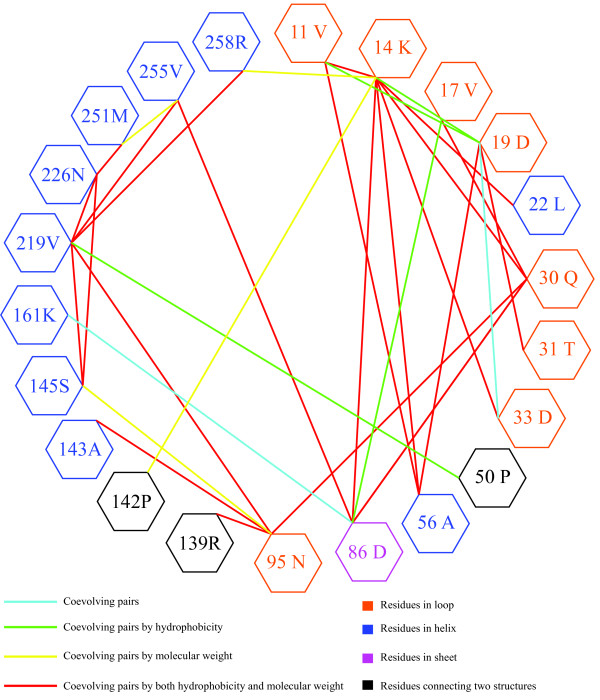
**Coevolutionary networks in the RbcL subunit of the three gymnosperm families**. Residues in the networks are sorted clockwise in an ascending order depending on the number of coevolutionary interactions that each amino acid residue establishes. The domains to which these amino acid sites belong are colour-coded. Nodes (amino acid residues) are connected through edges differently according to the nature and characteristics of their coevolution.

**Figure 5:**
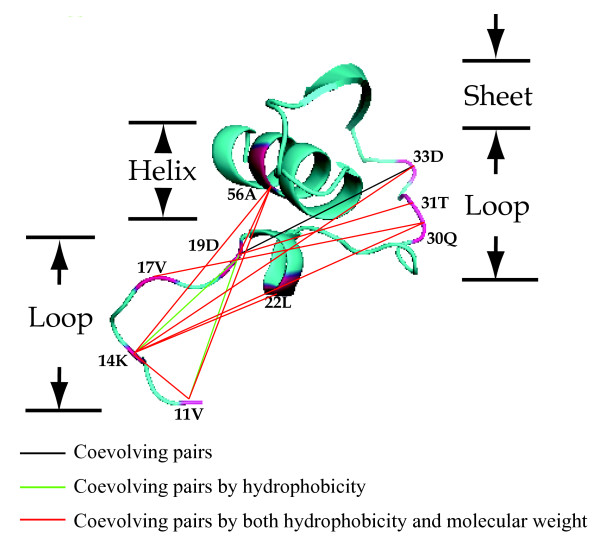
**Coevolving sites within the N-terminal structure of the RbcL subunit**. Amino acid residues involving in the coevolutionary network are highlighted red in the 3D structural diagram. And the residues are connected differently according to the nature and characteristics of their coevolution.

Analysis of coevolution in RbcL using CAPS identified 21 co-evolution groups (Figure 4). A coevolution group was defined to include all those sites that presented coevolution signal with all the other sites within the same group: if site A was coevolving with B, B with C and A with C, then all three sites were included within one coevolution group. The largest group (Figure 5) included four sites (11V, 14K, 19D, and 56A), while the smallest contained only two. The residues belonging to most of the co-evolving pairs presented significant correlations with their physicochemical properties, including hydrophobicity, molecular weight, or both of them. For example, four of the co-evolution pairs (11V/19D, 14K/19D, 11V/86D, and 50P/219V) detected among the 21 co-evolution groups exhibited correlated hydrophobicity; meanwhile, the other four pairs (14L/258R, 14K/142P, 95N/145S, and 255V/251M) correlated in their molecular weight variance.

## Discussion

### Adaptive evolution of the *rbcL *gene in the three gymnosperm families

The random-site models [30] were employed to examine the adaptive evolution of the *rbcL *gene in three gymnosperm families Podocarpaceae, Taxaceae and Cephalotaxaceae. Our results showed that most sites are under purifying selection, while a small part is under neutral evolution.

Recent improvements in the statistical models made it feasible to infer ancestral adaptive evolution events [31,32]. Using these models we found that the amino acid sites in the RbcL subunit of both the ancestor of Podocarpaceae and that of Taxaceae-Cephalotaxaceae had undergone adaptive evolution. By contrast, no adaptive evolution was detected in the ancestor of Cephalotaxaceae. Moreover, within Podocarpaceae, no positively selected site was found in the ancestral branch of *Podocarpus *I; however, two were detected in the branch leading to *Podocarpus *II (Figure 1). In Taxaceae, for the ancestor of *Taxus*, site 30 was identified as positively selected; but no such a site was found in the ancestor of *Torreya*. Totally, ten positively selected sites (A11V, Q14K, K30Q, G86D, S95N, V99A, I133L, S143A, L225I, and T262V) were identified in all the tests.

To fully understand the mechanism of the molecular adaptation of *rbcL *gene, both the structural and the functional significance of those ten positively selected sites need to be elucidated (Table 4). Seven (A11V, Q14K, K30Q, I133L, S143A, L225I, and T262V) of the positively selected sites are located at the interface of Rubisco subunits, whereas the other three (G86D, S95N, and V99A) are at the interface between Rubisco and its activase (Table 2 and 4).

**Table 4:** Functional roles of the amino acid sites under adaptive evolution

	Location			
			
Site	Domain	Interface	Functional roles	References
A11V	loop 1		To contribute to the holoenzyme thermal stability, catalytic efficiency, and CO_2_/O_2 _specificity	Kellogg and Juliano (1997) [3]
				
Q14K				Ott *et al. *(2000) [106]
			
K30Q	loop 2	LSU dimers		Du and Spreitzer (2000) [107]
			
I133L	βE			Spreitzer and Salvucci (2002) [36]
			
S143A	αD			Spreitzer *et al. *(2005) [64]
		
L225I	α2	LSUs and SSUs		Makowski *et al. *(2008) [33]
			
T262V	β4			

G86D	βC	Rubisco and its activase	To affect the contacts between the Rubisco and its activase	Du *et al. *(2003) [108]
			
S95N	βD			Portis (2003) [40]
				
V99A				Portis *et al. *(2008) [42]

All positively selected amino acid sites in Table 3 are included.

Most of the amino acid sites detected to be under adaptive evolution have been previously identified to have important functional roles. Of these adaptively selected sites, site 11 shows an adaptive substitution from Alanine to Valine. Valine has an additional methylene group (-CH_2_-) in comparison with Alanine, allows a stronger van de Waal binding with the other residues at the LSU_2 _C-terminus [33], tightening therefore the combination of LSU_2 _dimer [34]. In addition, lysine and glutamine present side-chains that are positively and negatively charged, respectively [35]. Therefore the replacement of Q by K at site 14, identified to have evolved under positive selection, may create extra ionic bonds with its proximal amino acid sites [36]. Importantly, site 30 is very close to site 28 [3], both possessing hydrophilic side-chains (Table 2). Kapralov and Filatov (2007) noted that site 28 is highly prone to fix amino acid replacements by positive selection in green plants [15]. Future examinations on sites 28 and 30 by using site-directed mutagenesis may unveil whether their hydrophilic side-chain substitutions have brought advantageous effects. The replacements at sites 133 and 143 may improve the stability of LSU_2 _dimer by modifying side-chain physicochemical characters [37,38]. Moreover, the nonsynonymous substitutions on sites 225 and 262 could contribute to the linkage of LSU and SSU subunits (Table 4). Recent genetic analyses of a hybrid Rubisco pointed to that the combination of LSU and SSU have effects on the holoenzyme expression [39].

Rubisco activase is involved in the opening of the closed state of the Rubisco form to release RuBP, producing the active enzyme form [40]. The physical contact between the Rubisco site 89 and the activase site 314 has been reported by Li *et al *(2005) [41]. Moreover, the negatively charged Rubisco site 93 is potentially complementary to the positively charged activase site 312 [42]. Hence, changes of the three neighbouring sites (G86D, S95N, and V99A) around sites 89 and 93 may affect the contacts between the Rubisco and its activase. In summary, positively selected sites identified in the *rbcL *gene of the three gymnosperm families are located either on the contact surface between Rubisco subunits or between the Rubisco and its activase. Nonsynonymous substitutions among them have great potentials to optimize the enzyme characteristics.

*RbcL *gene is located in the large single copy region of chloroplast genome [43,44]. It has been regarded as a region with no evidence for adaptive evolution [45], but recent research has brought this into question [15]. Currently, Ohno's model is frequently used to describe protein evolution and adaptation [46], which emphasizes the role that gene duplication plays in the adjustment of protein functions [47,48]. The "gene sharing" model stresses that it is at the single copy gene stage, namely before the occurrence of gene duplication, that functional genes have already adapted [49]. For instance, studies on the eye lens crystallins showed that the gene duplication indeed occurred after the protein had diverged from its ancestral function [50,51]. This study on the *rbcL *gene further unveils adaptive evolutionary processes in the single copy chloroplast gene. It has been known that for *rbcL*, only a few nonsynonymous substitutions, especially those at catalytic active sites on the LSU and SSU interface or between the Rubisco and its activase interface, can significantly change the Rubisco features (e.g. thermal stability, catalytic efficiency, and CO_2_/O_2 _specificity) [52]. Indeed, the positively selected sites we identified in the three gymnosperm families are all located at these positions. Adaptive substitutions at the sites, possibly generated during chloroplast DNA replication or repair [53], may impose great potential for optimizing the Rubisco functional/structural characteristics. As shown in Table 2, our results suggest that: i) mutations at most sites in *rbcL *(*p*_0 _= 89.2%, *ω*_0 _= 0.07) may be deleterious; ii) 5.8% of the substitutions probably have no significant effect on fitness; and iii) 2.3% of the replacements are likely to improve the Rubisco performance. It is therefore reasonable to postulate that the three gymnosperm families may have adaptively responded to habitat pressures by adjusting the Rubisco function/structure during their evolution.

### Historic adaptation of the *rbcL *gene under continual changing of CO_2 _concentration

The historic accumulation of nucleotide substitutions in the *rbcL *gene has supplied the Podocarpaceae species with differentiated Rubisco catalytic efficiency, which could contribute to their adaptive radiation into diverse ecological niches [54]. By contrast, the absence of *rbcL *adaptation in *Cephalotaxus *and *Torreya *may explain why their members tend to be locally distributed (Table 2).

Two positively selected sites were identified at the ancestral branch of Podocarpaceae and each was at the interface between the Rubisco and its activase (Table 2). The global CO_2 _concentration has been acutely changing since 170 Mya to 65 Mya [55]. Our results showed that the ancestor of Podocarpaceae diverged from Taxaceae-Cephalotaxaceae around 204 Mya; and its descendants started to diverge about 134 Mya [56]. From 170 Mya to 134 Mya, the *rbcL *variants in the ancestor of Podocarpaceae that could better adapt to the global reduction of CO_2 _concentration became consequently fixed (Figure 1, Table 2). Sage and Coleman (2001) has noted that increasing the expression level of the Rubisco and its activase is an effective solution for plants to respond to the reduction of CO_2 _concentration [57]. Here we further demonstrated that the simultaneous and inter-dependent adjustment of the two enzymes provides an alternative adaptive mode.

Many transgenic manipulations have been attempted to improve Rubisco's catalytic performance, since its property largely determines the maximum efficiency of photosynthesis in the use of light, water, and fertilizer N resources [58]. The function of Rubisco in terrestrial plants is identical. Evolutionary pressures seem to have driven it towards more efficient utilization of CO_2_, and recent analyses have indicated that the optimal performance of Rubisco is basically determined by CO_2 _concentration [59,60]. In this study, adaptive evolution of the *rbcL *gene has been detected in Podocarpaceae and *Podocarpus *I and II (Table 3), which implies that the Podocarpaceae species have undergone continual modification in their RbcL subunit for better fitness along with the changing atmosphere CO_2 _concentration. The positively selected sites identified (Table 3) may also benefit future studies for improving the Rubisco efficiency and for elucidating the interactions between the enzyme and its activase [61].

### Site-specific coevolution in RbcL subunit

To further understand the complex evolutionary patterns, we also analysed the inter-dependence among amino acid regions in the RbcL subunit. Adaptive evolution is only expected to occur on functionally and structurally meaningful amino acid sites where nonsynonymous substitutions are most likely to destabilise and hence to compromise organism's fitness [62,63]. Consequently, evolution of biological innovation is dependent upon the fixation of mutations close in the structure that can compensate the destabilising effects of innovative mutations. This may well explain the pattern we observe in the Rubisco, with many of the mutations that are under adaptive evolution and that co-evolve occurring at the interface of the protein dimer where stability is essential for the preservation of enzyme function and integrity. In support of the co-evolutionary hypothesis as a mean to fix innovative mutations in the RbcL subunit, previous research has shown that when multiple amino acid replacements are introduced, significant changes can occur in both enzyme catalytic efficiency and specificity [64]. In our co-evolutionary analyses, 21 co-evolution groups were identified within the RbcL subunit. In particular, evolutionary dependencies were recognized among sites belonging to different domains (Figure 5). Some correlated pairs (e.g. 251M and 255 V) were linearly proximal, whereas the others (e.g. 19D and 56A) were linearly distant but structurally proximal. Among the co-evolving sites, we also identified structurally and linearly distant sites (e.g. 14 K and 86D) (Figure 4 and 5). Although certain studies have attributed a biological meaning for the linearly proximal sites [65], many other reports elucidated that physical connections can be established between distant functional sites in the quaternary protein structures [66-69].

## Conclusions

This research presents substantial evidence that point to a complex adaptive process associated with the functional innovation of the Rubisco protein. This process involves a continuous checking of the structural and functional consequences of the fixation of novel mutations as well as the amelioration of the effects by such mutations through compensatory replacement events. Several other conditions related to the population genetics of the individuals have to be met in order for a compensatory mutation to be fixed before the individuals carrying the destabilising mutations are purged by selection. Among the possible hypothetical scenarios is the relaxation of the action of selection or the action of other buffering mechanisms. Species from both Taxaceae and Cephalotaxaceae are characterized by their small population sizes and clonality, which makes it feasible for genetic drift to act and facilitate the fixation of the mutations regardless of their immediate fitness consequences. Although this seems to be a possible scenario, several points remain to be investigated in future studies such as to assess the real inter-dependence among mutations through directed mutagenesis or to examine the strength of genetic drift at the population levels. Our research however opens exciting new avenues that may lead to a more complete understanding of the functional novelties in the Rubisco among gymnosperms.

## Methods

### Plant Sampling

Plant materials sampled for this investigation (Table 5), including *Cephalotaxus sinensis *(Rehder & E. H. Wilson) H. L. Li, *C. hainanensis *H. L. Li, *C. fortunei *Hook., *C. oliveri *Mast., *Taxus chinensis *(Pilg.) Rehder, *T. yunnanensis *W. C. Cheng & L. K. Fu, *T. mairei *(Lemee & H. Lev.) S. Y. Hu ex T. S. Liu, *Amentotaxus yunnanensis *H. L. Li, *A. argotaenia *(Hance) Pilg., *Torreya fargesii *var. *yunnanensis *(W. C. Cheng & L. K. Fu) N. Kang, *Pseudotaxus chienii *(W. C. Cheng) W. C. Cheng, *Podocarpus macrophyllus *(Thunb.) Sweet, *P. neriifolius *D. Don, *Dacrycarpus imbricatus *(Blume) de Laub., and *Dacrydium pierrei *de Laub., were collected from South China Botanical Garden, Chinese Academy of Sciences, Guangzhou, China. In addition, *Nageia nagi *(Thunb.) Kuntze was collected from the campus of Sun Yat-sen University, Guangzhou, China.

**Table 5:** Species and GenBank accession numbers for the *rbcL *gene sequences analysed in this study

Family	Genus	Species	GenBank Acc. No.
Taxaceae	*Taxus*	*Taxus mairei *(Lemee & H.Lev.) S. Y. Hu ex T. S. Liu	AY450856*
		*Taxus yunnanensis *W. C. Cheng & L. K. Fu	AY450857*
		*Taxus chinensis *(Pilg.) Rehder	AY450855*
		*Taxus baccata *L.	AF456388
		*Taxus cuspidata *Siebold & Zucc.	EF660720
		*Taxus sumatrana *(Miq.) de Laub.	EF660706
		*Taxus wallichiana *Zucc.	EF660717
		*Taxus canadensis *Marshall	EF660724
		*Taxus fuana *N. Li & R. R. Mill	EF660725
		*Taxus globosa *Schltdl.	EF660710
		*Taxus brevifolia *Nutt.	AF249666
		*Taxus x hunnewelliana *Rehder	EF660723
		*Taxus x media *Rehder	EF660722
	*Pseudotaxus*	*Pseudotaxus chienii *(W. C. Cheng) W. C. Cheng	AY450858*
	*Austrotaxus*	*Austrotaxus spicata *(R. Br. ) Compton	AF456385
	*Torreya*	*Torreya californica *Torr.	AY664858
		*Torreya taxifolia *Arn.	AF456389
		*Torreya nucifera *(L.) Siebold & Zucc.	AB027317
		*Torreya grandis *Fortune	EF660733
		*Torreya fargesii *Franch.	EF660735
		*Torreya fargesii *var. *yunnanensis *(W. C. Cheng & L. K. Fu) N. Kang	AY450861*
		*Torreya jackii *Chun	EF660734
	*Amentotaxus*	*Amentotaxus argotaenia *(Hance) Pilg.	AY450859*
		*Amentotaxus yunnanensis *H. L. Li	AY450860*
		*Amentotaxus formosana *H.L. Li	EF660708
Cephalotaxaceae	*Cephalotaxus*	*Cephalotaxus harringtonia *(Knight ex J.Forbes) K. Koch	EF660730
		*Cephalotaxus sinensis *(Rehder & E.H.Wilson) H. L. Li	AY450864*
		*Cephalotaxus koreana *Nakai	EF660726
		*Cephalotaxus wilsoniana *Hayata	AB027312
		*Cephalotaxus oliveri *Mast.	AY450865*
		*Cephalotaxus hainanensis *H. L. Li	AY450862*
		*Cephalotaxus griffithii *Hook. f.	EF660704
		*Cephalotaxus mannii *Hook. f.	EF660707
		*Cephalotaxus fortunei *Hook.	AY450863*
		*Cephalotaxus latifolia *Cheng & L. K. Fu	EF660712
		*Cephalotaxus fortunei *var. *alpina *H. L. Li	EF660714
		*Cephalotaxus lanceolata *K. M. Feng	EF660709
		*Cephalotaxus drupacea *Siebold & Zucc.	EF660716
Podocarpaceae	*Podocarpus*	*Podocarpus lawrencei *Hook. f.	AF249600
		*Podocarpus nivalis *Hook.	AF249619
		*Podocarpus gnidioides *Carriere	AF249607
		*Podocarpus totara *G. Benn. ex D. Don	AF307931
		*Podocarpus acutifolius *Kirk	AF249599
		*Podocarpus saligna *D. Don	AF249628
		*Podocarpus smithii *de Laub.	AF249629
		*Podocarpus hallii *Kirk	AF249609
		*Podocarpus nubigenus *Lindl.	AF249621
		*Podocarpus latifolius *(Thunb.) R.Br. ex Mirb.	AF249612
		*Podocarpus reichei *J. Buchholz et N. E. Gray	AF479879
		*Podocarpus cunninghamii *Colenso	AF249603
		*Podocarpus chinensis *Wall. ex J. Forbes	AF249602
		*Podocarpus fasciculus *de Laub.	AF249622
		*Podocarpus aff. pilgeri *MH6655	AF249624
		*Podocarpus aff. insularis *MH6612	AF249611
		*Podocarpus macrophyllus *(Thunb.) D. Don	AY450866*
		*Podocarpus aff. degeneri *MtLoftyBG	AF249627
		*Podocarpus elatus *R. Br. ex Endl.	AF249606
		*Podocarpus polystachyus *R. Br. ex Endl.	AF249626
		*Podocarpus lucienii *de Laub.	AF249615
		*Podocarpus polyspermus *de Laub.	AF249625
		*Podocarpus neriifolius *D. Don	AY450867*
		*Podocarpus longifoliolatus *Pilger	AF249614
		*Podocarpus novae-caledoniae *Viell.	AF249620
		*Podocarpus spinulosus *(Sm.) R.Br. ex Mirb.	AF249630
	*Nageia*	*Nageia nagi *(Thunb.) Kuntze	AY450868*
		*Nageia nankoensis *(Hayata) R.R. Mill	AF249649
	*Dacrycarpus*	*Dacrycarpus imbricatus *(Thunb.) de Laub.	AY450869*
		*Dacrycarpus dacrydioides *(Rich.) de Laub.	AF249597
	*Dacrydium*	*Dacrydium pierrei *de Laub.	AY450870*
Outgroup		*Pinus koraiensis *Siebold & Zucc.	NC_004677
		*Pinus thunbergii *Parl.	NC_001631
		*Welwitschia mirabilis *Hook.	NC_010654
		*Gnetum parvifolium *(Warb.) W. C. Cheng	NC_011942
		*Cycas taitungensis *C. F. Shen *et al.*	NC_009618
		*Ephedra equisetina *Bunge	NC_011954
		*Ginkgo biloba *L.	DQ069500

The sign * denotes sequences experimentally determined in the present study.

### Total genomic DNA extraction

Genomic DNA was extracted from leaves of individual trees by a modified cetyltrimethyl ammonium bromide (CTAB) method. 0.2 g fresh leaf tissue was ground to fine powder with a mortar and pestle in liquid nitrogen. The leaf powder was allotted equally into two Eppendorf tubes. One mL -20°C propanone was added to each tube. The tube was rocked gently for l min, centrifuged at 5,000 rpm for 10 min, and the supernatant was discarded; the procedure was repeated once. Each tube was added with 1 mL 60°C CTAB extraction buffer, mixed well, and incubated at 80°C for 4 hours. After incubation, 500 μl chloroform: isoamyl alcohol (24: 1) was added, mixed and then centrifuged at 13,000 rpm for 10 min. The top aqueous phase was removed to a new tube, added with 2/3 volume cold isopropanol, and mixed gently to precipitate DNA. DNA was dissolved in 70 μl TE buffer, and the quality was determined by 1% agarose gel electrophoresis.

### PCR amplification and DNA sequencing

PCR amplification of *rbcL *sequences was carried out in 100 μl volumes containing 50 mM KCl, 10 mM Tris-HCl (pH 8.0), 0.1% Triton X-100, 1.5 mM MgCl_2_, 0.2 mM each deoxynucleoside triphosphate, 2 U *Taq *DNA polymerase, 0.3 μM primer, 30 ng genomic DNA, and DNA-free water. The thermo-cycling program was set for 5 min at 95°C, 35 cycles of 1 min at 94°C, 2 min at 54°C, 3 min at 72°C, and 10 min at 72 °C. Negative controls where all reagents but DNA were added to the reaction mix were included in order to verify the absence of contamination. The forward and reverse primers were 5'-ATGTCACCACAAACAGAGACT-3' and 5'-CCTTCATTACGAGCTTGCACAC-3', respectively. PCR products and sizes were verified in agarose gels. The purified PCR products were sequenced directly in both forward and reverse directions. Three repeats of each fragment were determined to control for *Taq *polymerase errors.

### Phylogeny with timescale

DNA sequences of the coding regions obtained experimentally plus those retrieved from the public databases (Table 5) were multiple aligned using the MUSCLE software [70]. To improve the accuracy of phylogenetic inference, we excluded: i) multiple data from identical species, ii) sequences containing frame-shift mutations, and iii) ambiguously aligned regions. The appropriate DNA substitution model was identified by Modeltest v.3.7 package [27] via comparing 56 available models using the Akaike Information Criterion (AIC). The data set was also uploaded to the Datamonkey 2010 website for the estimation of the best fit model [28]. The tree topology inferred using the appropriate model for *rbcL *sequences was utilized as pre-defined tree in the adaptive and non-independent evolution analysis.

The following nodes within the phylogeny were chosen to constrain for a rate consistent with the known relationships: i) based on the *Cratonia cotyledon *fossils [71], the split of *Gnetum *and *Welwitschia *was constrained to 110 Mya (Node 72); ii) the good estimation of the split of *Taxus *and Cephalotaxaceae was constrained to 169 Mya (Node 66) [56]; iii) Node 75 was constrained to 225 Mya based on the earliest Pinaceae-type seed [72]; iv) Node 73 was constrained to 125 Mya based on the earliest *Ephedra*-type seed [73]. Following the suggestion of the authors of BEAST, we ran the empty alignment before the real data to avoid misspecification of dating and taxon sampling.

We applied BEAST to infer topology, branch lengths, and dates for the *rbcL *gene. As the relationships of Taxaceae and Cephalotaxaceae families have been historically under debates, the prior information on these two families was not given before the estimation. A normal distribution was applied over the estimating of the absolute ages via the MCMC process. BEAST runs of 4 × 10^7 ^generations, saving data every 1,000 generations, produced 40,000 estimates of dates under a Yule speciation prior and an uncorrelated relaxed clock [74] for the *rbcL *gene dataset. Convergence statistics was analyzed in Tracer, resulting in 36,000 post-burn-in trees. We used TreeAnnotator v. 1.5.3 [74] to produce maximum clade credibility trees from the post-burn-in trees and to determine the 95% probability density of ages for all nodes in the tree.

To illustrate the relationship between the ancestral adaptation and the concentration of CO_2 _in the atmosphere, the RCO_2 _value along with the time scale was mapped under the phylogeny (Figure 1) [55].

### Detection of positive and negative selection sites

Identification of adaptive evolution (positive selection) is fundamental to our understanding of the process of adaptation and diversifying selection. The general consensus is that nonsynonymous nucleotide substitutions (*d*_N_), whose alternatives leading to a change in the codon and its corresponding amino acid, can be scaled by the number of synonymous replacements (*d*_S_), which are nucleotide changes that only change the codon but not the amino acid and are consequently neutrally fixed and proportional to the divergence time between the sequences. Because the *d*_N _changed the amino acid sequence and protein function depending on its structure, this parameter is often under the filter of Natural selection. It follows consequently that the nonsynonymous-to-synonymous rates ratio (ω = *d*_N_/*d*_S_) can be considered as a stringent measure of selection [75,76]. Positive adaptive evolution occurs episodically during the evolution of proteins and this selection signal is generally swamped in a background signal of negative selection, which makes it difficult to robustly identify adaptive evolution. In order to identify signs of adaptive evolution we used two maximum-likelihood based models implemented in the CODEML program from PAML package version 4.1 [32], the random-site model and the modified branch-site model, for detecting the positive and negative selection sites within *rbcL *sequences among lineages. The random-site model allows the *ω *to vary among amino acid sites within the multiple sequence alignment and this parameter is estimated by maximum-likelihood following Goldman and Yang (1994) [77]. Conversely, the branch-site model (BSM) accounts for the variation in selective constraints among sites and lineages in the phylogeny synchronously. Within the BSM, we applied the modified model A test to reduce the false positive results as advised in the manual file of PAML version 4.1 [78]. In addition to the previous models, we also compared codon-based models that estimate one or several *ω *values for the different categories of codons. We conducted the likelihood ratio test (LRT) [79] to compare the different nested models (M0 vs. M3, M1a vs. M2a, M7 vs. M8, M8a vs. M8, and alternative test (*ω*_*2 *_estimated) vs. null test (*ω*_*2 *_= 1,fixed)) [80]. In the branch-site models, the branches were selected to testify whether the species have a bigger opportunity to undergo an episodic adaptive evolution along with the acutely changing atmospheric CO_2 _concentration (Figure 1, grey regions). The following seven lineages were selected: (i) Taxaceae-Cephalotaxaceae, (ii) *Taxus*; (iii) *Torreya*; (iv) Podocarpaceae; (v) *Podocarpus *I; (vi) *Podocarpus *II; and (vii) Cephalotaxaceae. To address the problem of multiple comparisons, the Bonferroni correction was employed during the continuous checking with the A-A1 models [29].

The multiple sequence alignment (MSA) was also submitted to the Selecton website [81] for the comparison between empirical models and mechanistic empirical combination (MEC) models. Moreover, the ancestral states of the *rbcL *sequences were reconstructed via the DAMBE package [82] and the ASR module on the Datamonkey website [28,83]. The offspring sequences were compared with the ancestral sequences on each node. And the sequence was submitted to European Bioinformatics Institute (http://www.isb-sib.ch/ Swiss-model) for predicting the three-dimensional structure of the RbcL subunit.

### Identification of intra-protein coevolutionary pattern

To understand the broad implications of the amino acid replacements in the RbcL subunit we conducted an analysis of the evolutionary dependencies among sites to identify functional/structural dependencies among residues. If two amino acid sites were under adaptive evolution and these sites were co-evolving, this may indicate their functional/structural dependency. Intra-protein co-evolution in *rbcL *was tested via the program CAPS [20]. This algorithm implemented in this program takes the phylogenetic dependencies into account and correct them [84] and has been reported to outperform other approaches [85].

Broadly, CAPS compares the correlated variance of the evolutionary rates at two sites corrected by the time since the divergence of the two sequences. The significance of the results was evaluated by randomization of pairs in the alignment, calculation of their correlation values, and comparison of the real values with the distribution of 10,000 randomly sampled values. The step-down permutation procedure was employed to correct for multiple tests and non-independence of data [86]. An alpha value of 0.001 was applied to minimize type I error. The correlated variability between amino acid sites was weighted by the level of substitutions per synonymous site in order to normalize parameters by the time of sequence divergence [87].

## Competing interests

The authors declare that they have no competing interests.

## Authors' contributions

LS carried out the molecular evolution analysis, participated in the sequence alignment and drafted the manuscript. MAF contributed to the analysis tools and drafted the manuscript. BL participated in the design of the study and helped to draft the manuscript. LG helped to draft the manuscript. BW performed the statistical analysis. TW and YJS conceived of the study, and participated in its design and coordination and helped to draft the manuscript. All authors read and approved the final manuscript.

## Reviewers' comments

### Reviewer's report 1

Prof. Christian Blouin (nominated by W Ford Doolittle), Dalhousie University Halifax, Nova Scotia, Canada

This reviewer provided no comments for publication.

### Reviewer's report 2

Dr Endre Barta (nominated by Sandor Pongor), International Centre for Genetic Engineering and Biotechnology, Trieste, Italy

Gymnosperm plants played an important role in Earth's flora, especially in the prehistoric ages. In this manuscript, the authors use elegant molecular evolutionary analyses to answer some open questions about the evolutionary processes tailoring the chloroplast-coded *rbcL *gene during the adaptation to the changing CO_2 _concentration in the atmosphere. The authors use *rbcL *coding sequences from three gymnosperm families. The *rbcL *is a very specific and very constrained protein, coded in the chloroplast genome and also being in complex with the *rbcS*, which is coded in the nucleus. The three gymnosperm families are good subjects for this analysis because i) they represent almost 14% of the gymnosperm diversity and can be found globally on the Earth, ii) we have fossil records allowing to constrain the phylogenetic tree timescale.

The authors present a robust evolutionary analysis based on the multiple alignment of *rbcL *coding sequences from different gymnosperm species. They found that a complex adaptation process occurred during the evolution of these taxa. They also discussed the structural and functional consequences of these processes and concluded that certain compensatory replacement mutations could play important role in the fixation of the functionally novel mutations. The analysis is very sound and in most cases based on different methods and models. The basic idea and the results can be interesting for the broader community.

I have only some theoretical questions and three minor technical comments.

Questions:

Are there any known examples for the same compensatory mutation pairs from other plant species (i.e. evidence for convergent evolution)?

***Authors' response: ****Recent report of the modification on both large and small subunits of Rubisco enzyme in *Flaveria *(Asteraceae) might be an example for the similar patterns *[88]*. The two subunits are under selection during the evolution from C*_*3 *_*to C*_*4 *_*photosynthesis. This pattern may be an evidence for convergent evolution under ecological pressures. However, the compensatory mutation pair is not coincided in the study*.

How could the geographical isolation of different populations influence the results of this study? Are there any samples (*rbcL *sequences) from geographically well separated plants from the same species? Do you expect any polymorphisms at any replacement site in the small populations of these gymnosperm species?

***Authors' response: ****If we take the geographical CO*_*2 *_*variation into account, the geographical isolation of different populations will significantly influence the results of the present study. As has been reported previously, the *rbcL *gene has undergone adaptive evolution during the radiation in the Hawaiian endemic genus *Schiedea, *which demonstrates that *rbcL *gene evolved under strong positive selection impacted by the geographical isolation *[13]*. Nonetheless, the present research is mainly focused on the relations above species level, so the samples (*rbcL *sequences) from the same species are excluded in the analyses. The polymorphisms at those replacement sites probably exist in the small populations of these gymnosperm species*.

The inferred tree topology and the taxonomic classification of the genera in Taxaceae seem to be different. How can you explain this?

***Authors' response: ****Since its lower evolutionary rate, the *rbcL *gene has certain limitations on the deeper phylogenetic levels (e.g. at the genus level) *[89]*. On the other respect, the molecular adaptation in *rbcL *gene per se also has impact on the inferring of the phylogenetic trees *[17]*. The above two factors may be the explanation for the disagreement between the inferred tree topology and the taxonomic classification*.

Is it possible to deduce from this analysis the ancestral sequence of the *rbcL *gene characteristic for the different nodes?

***Authors' response: ****Positive answer. However, more experimental data is required for the inference of the *rbcL *gene characteristic even though the inferring of the ancestral sequence from the current ones is of statistical efficiency *[90,91]. *We believe that much more work have been left for the further research after our computational estimation*.

Comments:

Reading the abstract at a first glance, it is not clear what is the relation between Rubisco and *rbcL*. Clarifying this would help the readers who are not familiar in plant biology.

***Authors' response: ****We agree with this remark and changed the sentence accordingly. One sentence has been added into the abstract especially for the introduction of the relation between Rubisco and *rbcL *gene*.

It is very difficult to review the tables in general, and especially the Table 3. Using grids, or re-structuring them would help a lot.

***Authors' response: ****We agree with this remark and re-structured Table *3*(new Table *2) *accordingly*.

Referencing Figure 4 is before the first reference to Figure 3, and no reference for Table 1 in the text.

***Authors' response: ****We appreciate the constructive comment. The order of referencing figures has been re-checked. Since Table *1*shows the original plant materials of this research, which cannot be omitted, we changed its appearance from the first table (former Table *1) *into the last one (new Table *5).

### Reviewer's report 3

Dr Nicolas Galtier, CNRS-Université Montpellier II Laboratoire "Genome, Populations, Interactions, Adaptation", Montpellier, France

This manuscript analyses the molecular evolution of the essential *rbcL *gene in three gymnosperm families. The functional relevance of amino-acid sites detected as positively selected or co-evolving is discussed. Here are my major comments:

The text is quite affirmative regarding divergence dates, and their relationship with atmospheric CO_2 _abundance. I am not sure that molecular dating is that trustable, even with the use of clock-relaxed models, as illustrated by many controversies in the recent literature (e.g. Graur & Martin 2004 Trends Genet, Douzery *et al. *2004 PNAS, Peterson *et al. *2004 PNAS, Roger & Hug 2006 Philos Trans, Emerson 2007 Syst Biol), owing to paleontological uncertainty and tricky rate/time decoupling [92-96]. Some prudence would appear required here, and the uncertainty of date estimates could be discussed. This is especially true knowing that the uncorrelated model in BEAST was used here, an approach which was criticized in the recent past (Lepage *et al. *2007 Mol Biol Evol) [97].

***Authors' response: ****Due to the paleontological uncertainty and trick rate/time decoupling, it is still an unresolved scientific theme whether the modern molecular clock has the ability to reconcile the fossil evidence and the time estimation *[98]*. However, as indicated in many other literature (e.g. Welch & Bromham 2005 Trends Ecol Evol, Ho 2007 J Avian Biol, Ho 2009 Biol Lett), lots of recent methodological advances have been carried out focusing on the topic *[99-101]*. Specifically, although correlated model (also known as correlated-rates model, or CR model) outperforms the uncorrelated model (also known as independent-rates model, or IR model) in the instance provided by Lepage et al (2007) *[97]*, several authors have noticed that the uncorrelated model is better than the correlated model during estimating the dynamics of evolutionary rates in other instances *[22,102-104]*. Moreover, Zhong et al. (2009) argued quite recently that the uncorrelated model is superior to the correlated model in guesstimating the episodic rate acceleration in ancestral plant lineages *[105]*. Collectively, all the above conclusions indicate that the modern molecular clock relied on uncorrelated model is applicable for our present study on the gymnosperm plants*.

The reason for species sampling in this study is not obvious. Just three families were (thoroughly enough) sampled, when the focus of the study is on *rbcL *adaptation during the > 200 Mya of gymnosperm evolution. A more balanced sampling across gymnosperm families might help corroborate some of the results reported here.

***Authors' response: ****The three families (Podocarpaceae, Taxaceae and Cephalotaxaceae) represent over 14% of the gymnosperm diversity and can be found globally on the Earth *[23,24]*. Moreover, reliable fossil records can be obtained to calibrate molecular clock for dating the time of the phylogenetic trees *[56]*. So the thoroughly enough sampled species in the three families could partially represent adaptive and coevolutionary patterns of *rbcL *gene in the related gymnosperms under geological timeline. And we also believe that further research including other families will shed new lights on the big thesis*.

Along the same lines, it would be good to know whether the sites identified as positively selected or coevolving in gymnosperms behave similarly in angiosperms (and perhaps other groups of plants), for which a huge database of *rbcL *sequences is available.

***Authors' response: ****As far as we can see, the atmospheric CO*_*2 *_*concentration is one important factor (also known as ecological pressure) related to the adaptation of Rubisco enzyme *[14,60]*. Nevertheless, other factors also have impact on the evolution of this enzyme. For instance, the C*_*3*_*/C*_*4 *_*photosynthesis in angiosperms have effects on the modification of *rbcL *gene *[5]*and *rbcS *gene *[88]*. The comparison analyses merely along the identical timeline, ignoring other ecological pressures, may mislead the conclusions. Since the above reasons, we only focus our sampling on the present families*.

The discussion emphasizes potential adaptive processes, in possible connection to CO_2 _availability across time. I note that if *rbcL *evolution was related to atmospheric CO_2 _variations, we would expect adaptive evolution to occur simultaneously in contemporary branches of the tree, in line with sudden RCO_2 _changes. Such a pattern is not clearly detected, so I wonder what in the data makes the author link *rbcL *evolution to atmospheric CO_2 _concentration, especially knowing that the adaptative signal is not prominent.

***Authors' response: ****The ability to undertake adaptive evolution depends on several factors. Gymnosperm plants played an important role in Earth's flora, especially in the prehistoric ages. This implies that the species of the three gymnosperm families have a higher feasibility to undergo adaptation in the prehistoric branches. Along with the rising of angiosperms, members from the contemporary branches of gymnosperm plants are characterized by their small population sizes, which make them feasible for genetic drift. The current analysis results and the biological background drew us a big imagination of the ancestral *rbcL *gene adaptation associating with the variations of the atmospheric CO*_*2 *_*concentration*.

## List of abbreviations used

Rubisco: ribulose-1, 5-biphosphate carboxylase/oxygenase; RuBP: D-ribulose-1, 5-bisphosphate; cp: chloroplast; RbcL: large subunit of Rubisco enzyme; RbcS: small subunit of Rubisco enzyme; LSU: large subunit; SSU: small subunit; MSA: multiple sequence alignment; Mya: million years ago; MRCA: the most recent common ancestor; K/T extinction: Cretaceous-Tertiary extinction; UCLD: uncorrelated lognormal model.
